# *In Vitro* Characterization of Guanylyl Cyclase BdPepR2 from *Brachypodium distachyon* Identified through a Motif-Based Approach

**DOI:** 10.3390/ijms22126243

**Published:** 2021-06-10

**Authors:** Maria Duszyn, Brygida Świeżawska-Boniecka, Aloysius Wong, Krzysztof Jaworski, Adriana Szmidt-Jaworska

**Affiliations:** 1 Chair of Plant Physiology and Biotechnology, Faculty of Biological and Veterinary Sciences, Nicolaus Copernicus University in Toruń, Lwowska St. 1, PL 87-100 Torun, Poland; swiezawska@umk.pl (B.Ś.-B.); jaworski@umk.pl (K.J.); asjawors@umk.pl (A.S.-J.); 2 Department of Biology, College of Science and Technology, Wenzhou-Kean University, 88 Daxue Road, Ouhai, Wenzhou 325060, China; alwong@kean.edu; 3 Zhejiang Bioinformatics International Science and Technology Cooperation Center, Wenzhou-Kean University, Ouhai, Wenzhou 325060, China

**Keywords:** guanylate cyclase, guanylyl cyclase, moonlighting proteins, 3′,5′-cyclic guanosine monophosphate, cGMP, PepR2, *Brachypodium distachyon*

## Abstract

In recent years, cyclic guanosine 3′,5′-cyclic monophosphate (cGMP) and guanylyl cyclases (GCs), which catalyze the formation of cGMP, were implicated in a growing number of plant processes, including plant growth and development and the responses to various stresses. To identify novel GCs in plants, an amino acid sequence of a catalytic motif with a conserved core was designed through bioinformatic analysis. In this report, we describe the performed analyses and consider the changes caused by the introduced modification within the GC catalytic motif, which eventually led to the description of a plasma membrane receptor of peptide signaling molecules—BdPepR2 in *Brachypodium distachyon*. Both in vitro GC activity studies and structural and docking analyses demonstrated that the protein could act as a GC and contains a highly conserved 14-aa GC catalytic center. However, we observed that in the case of BdPepR2, this catalytic center is altered where a methionine instead of the conserved lysine or arginine residues at position 14 of the motif, conferring higher catalytic activity than arginine and alanine, as confirmed through mutagenesis studies. This leads us to propose the expansion of the GC motif to cater for the identification of GCs in monocots. Additionally, we show that BdPepR2 also has in vitro kinase activity, which is modulated by cGMP.

## 1. Introduction

Guanosine 3′,5′-cyclic monophosphate (cGMP) is a key signaling molecule that is involved in signal transduction and controls different physiological responses and processes in numerous prokaryotic and eukaryotic cells. The concentration of cyclic GMP is regulated by guanylyl cyclases (GCs) that catalyze the synthesis of cGMP from GTP and phosphodiesterases (PDEs) that hydrolyze cGMP to GMP. The role and importance of cGMP and GC in mammals and prokaryotes was known for a long time, while their presence in plants was questionable, as they show less activity in vitro than in animal cells [1,2]. However, in recent years, an increasing number of reports on proteins with GC activity have appeared, which indicates that they play important roles in signal transduction and in several physiological processes in plants [3,4]. The cGMP-dependent signaling pathway is involved in root gravitropism of *Glycine max* [5], phytochrome-controlled induction of flowering of *Pharbitis nil* [6], and regulation of *Arabidopsis thaliana* seed germination [7]. Studies showed that proteins with confirmed in vitro GC activity are involved in plant growth (AtBRI1 [8], AtPSKR1 [9,10]), pollen tube guidance (AtDGK4 [11]), and response to pathogens (AtPepR1 [12], HpPepR1 [13], OsWAKL21.2 [14], SlGC17, and SlGC18 [15]).

Significant progress was made in identifying and characterizing new GCs after deducing the pattern search motif of the catalytic center of GCs. The motif includes a highly conserved 14-amino-acid catalytic motif, and it was the first alignment of designated catalytic regions from prokaryotes and eukaryotes [16]. In subsequent research, this motif was the basis for the search for new plant GCs. Moreover, with increasing knowledge, the motif was steadily improved, and new amino acids were added, which enables us to distinguish between strict and relaxed GC motifs [17,18].

In this study, we investigated PepR2 from *Brachypodium distachyon* (BdPepR2), which belongs to the leucine-rich repeat receptor-like protein kinase family and is the orthologs of the PepR1 from *A. thaliana* and *Hippeastrum hybridum,* which were experimentally confirmed to be functional GCs [12,13]. Based on in vitro analysis, the recombinant BdPepR2 protein was determined to have GC activity. The previously described GC catalytic center contains conserved residues lysine or arginine [KR] at position 14, which are involved in stabilizing the transition to cGMP. In the case of BdPepR2, methionine [M] is at this position in the center; [M] was never considered or tested as one of the key amino acids for GC activity. However, our results indicated that mutating this amino acid to arginine [R] or alanine [A] lowers GC activity. This result implies first that [M] at position 14 of BdPepR2 is optimal for GC activity, and second that it is worth expanding the GC motif to identify new cyclases, especially from monocotyledonous plants. To the best of our knowledge, BdPepR2 is only the third active GC that contain a highly conserved 14-aa search center in the GC catalytic site reported from monocotyledonous plants, after HpPepR1 [13] and OsWAKL21.2 [14]. Additionally, we show that BdPepR2 also has in vitro kinase activity, which is modulated by cGMP, indicating that it is a twin-domain molecule.

## 2. Results and Discussion

### 2.1. Sequence Analysis of BdPepR2

**Figure 1:**
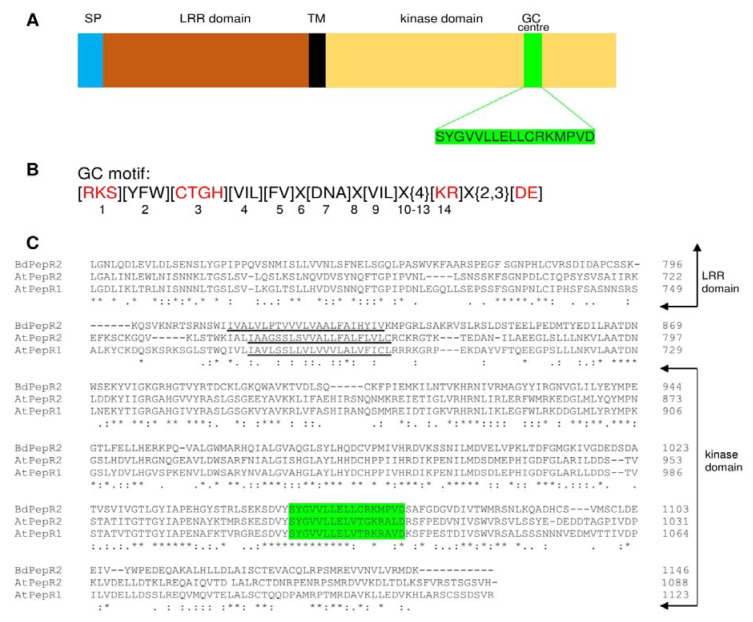
Structural features of GC catalytic domain of BdPepR2. (**A**) Representation of domain organization of BdPepR2 containing a signal peptide (SP), leucine-rich repeats (LLRs), transmembrane domain (TM), and GC center embedded in kinase domain. (**B**) Fourteen amino acid long original search motif; red amino acids are functionally assigned residues of the catalytic center. Residue in position 1 can form hydrogen bond with purine; residue in position 3 confers substrate specificity; residue in position 14 stabilizes transition state from GTP to cGMP; amino acid [D or E] at 2–3 residue downstream from position 14 participates in Mg^2+^/Mn^2+^-binding. (**C**) Amino acid sequence alignment of BdPepR2 (XP_003571653.1), AtPepR1 (OAP14914.1), and AtPepR2 (OAP12577.1) using the Clustal Omega. Predicted protein domains are indicated on the right side, TM is underlined, GC motif is highlighted in green. An * (asterisk) indicates positions that have a single, fully conserved residue. A: (colon) indicates conservation between groups of strongly similar properties. A. (period) indicates conservation between groups of weakly similar properties.

The amino acid sequence of BdPepR2 is referred to in the NCBI database as a putative leucine-rich repeat receptor-like protein kinase PEPR2 (NCBI: XP_003571653.2). Analysis of the 3526-bp ORF sequence corresponding to the 1146-aa polypeptide using the Blast program demonstrated similarity with other receptor-like protein kinases. The amino acid sequence of BdPepR2 exhibited only 38.17% amino acid identity with AtPepR2, and 36.26% amino acid identity with AtPepR1, a protein with confirmed GC activity from *A. thaliana* [12] (as illustrated in Figure 1C). *B. distachyon* is a monocotyledonous plant, whereas *A. thaliana* is a dicotyledonous plant; thus, divergences in the amino acid sequences of these proteins may result from their separate evolutionary development. The *BdPepR2* gene is predicted to encode a protein with 1146 amino acid residues with domain organization typical of LRR receptor-like kinases (RLKs) and highly conserved LRR domains [19] (as illustrated in Figure 1A). The N-terminus contains a hydrophobic secretion signal followed by an extracellular domain with 24 tandem copies of a 24-residue LRR (residues 121 to 745). A single transmembrane domain (residues 810 to 832) is predicted to separate the extracellular domain from an intracellular Ser-Thr kinase domain (residues 870 to 1146) with an embedded GC motif [16]. The mentioned motif [RKS] [YFW] [GCTH] [VIL] [FV] x(3) [VIL] x(4) [KR] is present in the catalytic center of guanylyl cyclases of several *A. thaliana* proteins, including brassinosteroid receptor AtBRI1 [8], wall-associated kinase like 10 AtWAKL10 [20], Pep1 receptor AtPepR1 [12], phytosulfokine receptor AtPSKR1 [9], nitric oxide-binding GC AtNOGC1 [21], plant natriuretic peptide receptor AtPNP-R1 [22], *H. hybridum* HpPepR1 [13], and 99 GC candidates in *Solanum lycopersicum* [15].

As presented in Figure 1B, the red amino acids at positions 1, 3 and 14 within the search motif are functionally assigned residues [16]. In the case of BdPepR2, the serine [S] localized at the 1 position is predicted to form a hydrogen bond with guanine, the glycine [G] at position 3 confers substrate specificity for GTP, and the amino acid at position 14 binds to the phosphate acyl group and stabilizes the transition state from GTP to cGMP. In BdPepR2, there is a methionine [M] in this position, and to the best of our knowledge, such an amino acid was never studied as a potential amino acid in the GC domain. Therefore, our objective was to verify whether the functional core motif could be extended by the addition of methionine. The identified BdPepR2 protein sequence also contains aspartic acid [D], a residue responsible for interaction with Mg^2+^/Mn^2+^ ions located two amino acids behind the conserved motif [17].

### 2.2. Purification of BdPepR2 Recombinant Protein and In Vitro GC Activity

The truncated 942-bp fragment of the BdPepR2 cDNA, including full kinase domain with the GC motif, was cloned into the pGEX-6P-2 vector in frame with a glutathione S-transferase (GST) tag and expressed in *E. coli* BL21 cells as a GST-BdPepR2 recombinant protein. The molecular mass of the 313-aa-long BdPepR2 polypeptide was predicted in silico to be 35.35 kDa, and the isoelectric point was predicted to be 5.76 (http://web.expasy.org/compute_pi; accessed on 9 January 2021). The recombinant BdPepR2 protein was purified and used for GC enzymatic activity determination. Affinity chromatography enabled the purification of the GST-BdPepR2 fusion protein as a clear main 60 kDa band. Removing the GST tag from the fusion protein by digestion with PreScission protease resulted in the appearance of one distinct protein measuring approximately 36 kDa, which corresponded well with the predicted molecular mass of 35.35 kDa for the truncated BdPepR2 protein (as illustrated in Figure 5B).

**Figure 2:**
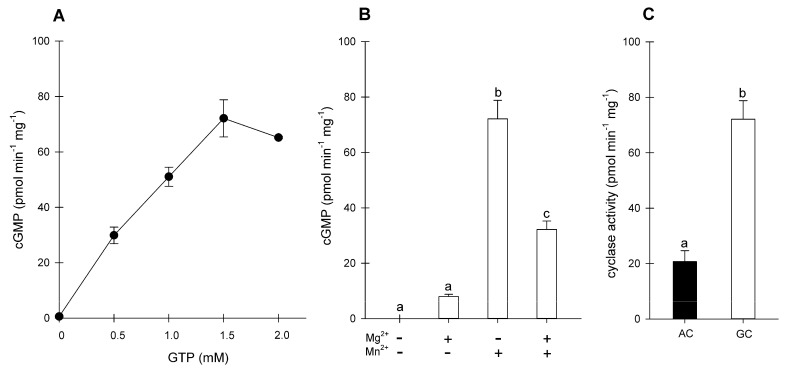
Enzymatic activity of recombinant BdPepR2. (**A**) Recombinant BdPepR2 activity in response to various concentrations of GTP. Reaction mixture contained 50 mM Tris/HCl buffer (pH 7.5), 5 mM MnCl_2_, GTP (0.5–2 mM), and 5 μg of purified protein (without GST-tag) in a final volume of 100 μL. (**B**) Determination of BdPepR2 cofactor specificity. Reaction mixture contained 50 mM Tris/HCl buffer (pH 7.5), 5 mM MnCl_2_ and/or 5 mM MgCl_2_, 1.5 mM GTP and 5 μg of purified protein (without GST-tag) in a final volume of 100 μL. (**C**) Determination of BdPepR2 substrate specificity. Reaction mixture contained 50 mM Tris/HCl buffer (pH 7.5), 5 mM MnCl_2_, 1.5 mM GTP or 1.5 mM ATP, and 5 μg of the purified protein (without GST-tag) in a final volume of 100 μL. Data are mean values (n = 3), and error bars show standard error of mean. Statistical analysis was performed by one-way ANOVA followed by a Tukey–Kramer multiple comparison test. Different letters indicate significantly different data.

**Figure 3:**
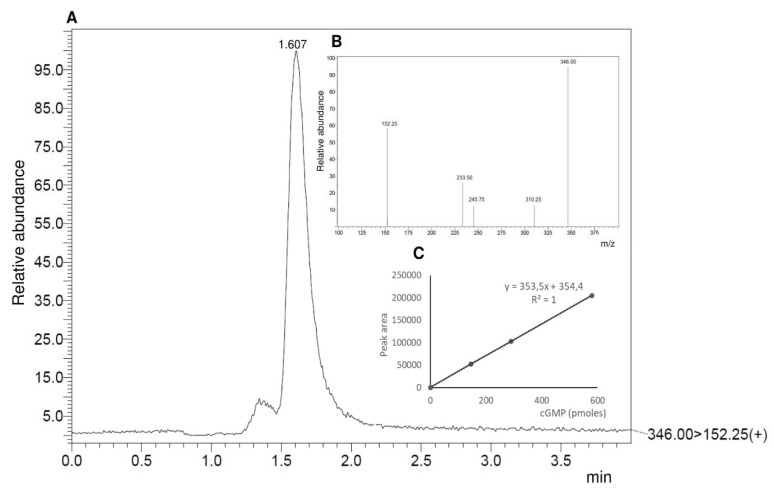
Detection of cGMP generated by BdPepR2 by LC-MS/MS. (**A**) Determination of GC activity of the BdPepR2 protein by LC-MS/MS. Ion chromatogram of cGMP was generated from a reaction mixture containing 5 µg of purified protein and GTP as a substrate in the presence of 5 mM Mn^2+^. (**B**) Inset showing parent cGMP ion at *m*/*z* 346.00 [M + H]+ and corresponding fragmented daughter ion at *m*/*z* 125.25 [M + H]+. Fragmented product ion was used for quantitation. (**C**) Inset showing the cGMP calibration curve performed with 0–0.58 pmoles of pure cGMP on the column.

Subsequently, the purified intracellular domain of the BdPepR2 protein was tested for its ability to convert GTP substrate to cGMP in the presence of magnesium and/or manganese ions as cofactors. The maximal BdPepR2 activity was reached at 1.5 mM GTP 15 min after starting the reaction, and the generated level of cGMP was 72.1 pmol mg protein^−1^ min^−1^ (±9.48) (as illustrated in Figure 2A and Figure 3). The kinetic parameters determined for BdPepR2 were a Vmax of 112.1 fmole min^−1^ ug^−1^ protein and a Km value of 1.189 mM. The BdPepR2 GC activity was higher compared to that of the results previously determined for other plant guanylyl cyclases, e.g., HpPepR1 (17 pmol cGMP mg protein^−1^ min^−1^) [13], AtPepR1 (∼2.5 pmol mg^−1^ min^−1^) [12], and AtBRI1 (∼4.3 pmol mg^−1^ min^−1^) [8]. However, compared to that of the activity described for PnGC1 (78.1 pmol mg^−1^ min^−1^) [23] and for HpGC1 (600 pmol mg^−1^ min^−1^) [24], the observed maximum GC activity of BdPepR2 was similar or considerably lower. At present, there are two groups of GCs: the first with the canonical GC domains, that often appears as a stand-alone molecule, and in the second group, the cyclase domain is part of a larger, multidomain protein complex. Of the proteins mentioned above, only PnGC1 and HpGC1 do not have a complete motif and are canonical GCs. The remaining proteins are so-called moonlight proteins. In addition to the GC domain, these proteins also have kinase domains, many of which have confirmed in vitro activity that presumably may affect GC activity. Moonlighting proteins have multiple tasks and have significant roles that contribute to the whole plant [4]. Therefore, it is possible that the GC activity of these proteins is lower than that of PnGC1 and HpGC1, which are presumed cytoplasmic proteins with unknown activity other than GC. Furthermore, such large differences in GC activity levels may be associated with the sensitivity of the measurement methods utilized to determine cGMP concentration. Over the past years, various methods were used, including [3H] cGMP radioimmunoassay, [125I] the cGMP assay system, cGMP enzyme immunoassay (EIA) system, and the high-resolution detection method LC-MS/MS. Due to the use of different methods and their different means of optimization, it is difficult to compare the activities of the guanylyl cyclases present in plants. However, regardless of the method used, the level of GC activity, and thus, the level of cGMP in plants, is always lower than that recorded for animal cells [25]. The main reason for this difference is that higher GC activities might require cofactors (e.g., calcium ions, chaperones, and coproteins) or unknown posttranslational modifications.

In the BdPepR2 sequence, aspartic acid [D] is located two amino acids behind the conserved motif. Based on the findings reported in the available literature, [DE] residues at this position are responsible for interactions with Mg^2+^/Mn^2+^ ions [17]. Thus, we predicted that divalent cations are the cofactors necessary for the CG activity of BdPepR2, and we tested whether it is Mn^2+^-dependent or Mg^2+^-dependent. The results showed that the studied GC domain of BdPepR2 has a higher affinity for Mn^2+^ than Mg^2+^ ions (as illustrated in Figure 2B). The same situation was observed in the case of previously examined AtPepR1-GC [12], PnGC1 [23], and AtNOGC1 [21]. Other recombinant GCs, such as AtWAKL-10 [20], AtBRI1 [8], AtGC1 [16], AtPNP-R1 [22], and HpPepR1 [13], exhibited a preference for Mg^2+^ over Mn^2+^.

The catalytic centers of plant GCs and adenylyl cyclases (ACs) share a high level of amino acid similarity and differ only in the residue at position 3, which is responsible for substrate recognition [16]. We tested BdPepR2 for AC activity in the presence of 1.5 mM ATP as the substrate. Mass spectrometry analysis showed more than fourfold lower BdPepR2 AC activity (~16.26 pmol mg protein^−1^ min^−1^), clearly indicating that the analyzed protein has GTP preference (as illustrated in Figure 2C).

### 2.3. Mutational Analysis

**Figure 4:**
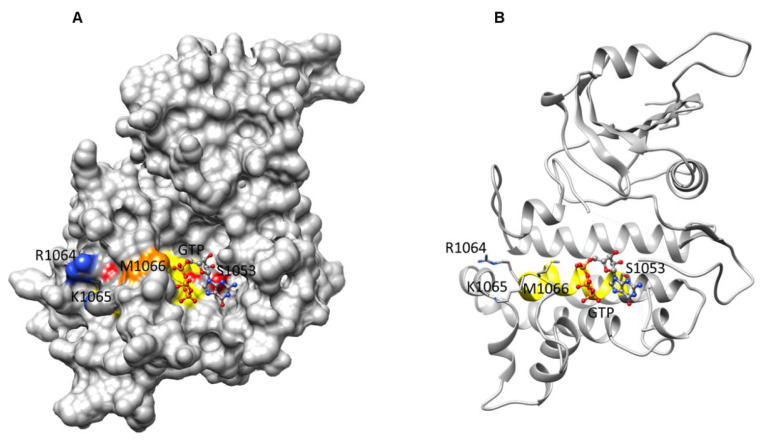
Docking of GTP at the GC center of BdPepR2 and interaction of GTP with key residues at catalytic center (yellow) is shown as (**A**) surface and (**B**) ribbon models, respectively. Functional residues at positions 1 (S1053, red) and 14 (M1066, orange) of the motif and neighboring R1064 (position 12) and K1065 (position 13) facing away from the GC center are shown.

In the GC catalytic center of BdPepR2 [1052-SYGVVELLCRKMPVD-1070], all amino acids except [M] were confirmed to be essential for cyclase activity [17,18]. In the conserved model at this position, [KR] is preferable over [M] [18]. However, structural analysis suggests that in the case of BdPepR2, [M] can assume the role of [KR] with positive docking affinity of GTP at the GC center. Molecular docking of GTP to the catalytic center of the BdPepR2 [Met834→Lys1146] model elucidated the substrate pose, orientation, and interactions with the key residues serine, arginine, and methionine of the GC center (as illustrated in Figure 4). Homology model predictions also showed that neighboring R1064 (position 12) and K1065 (position 13) were unlikely to bind GTP because they were facing away from the GC center (as illustrated in Figure 4).

**Figure 5:**
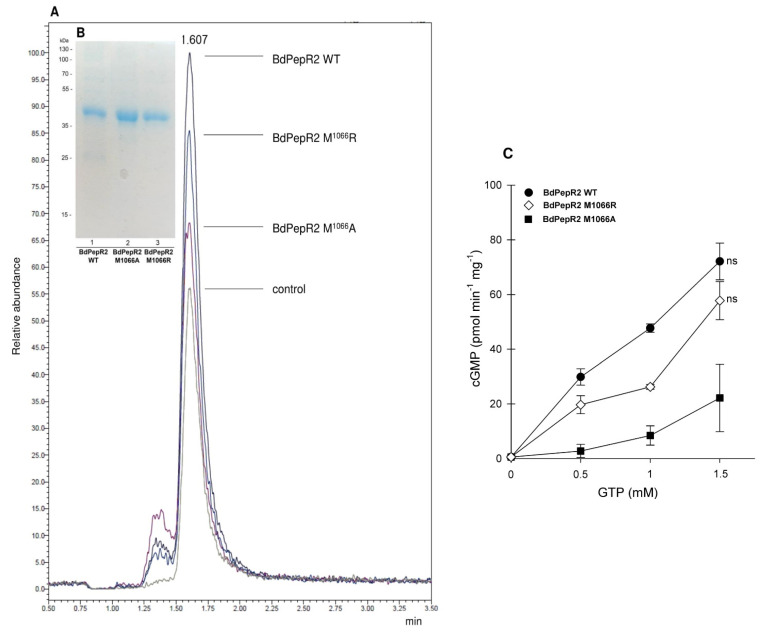
Detection of cGMP generated by BdPepR2 WT, BdPepR2 M1066A, and BdPepR2 M1066R by LC-MS/MS (**A**) Four superimposed ion chromatograms of cGMP generated by 5 µg recombinant BdPepR2 WT (black), BdPepR2 M1066R (blue), BdPepR2 M1066A (purple), and control (no protein, grey) after 15 min in the presence of 5 mM Mn^2+^. (**B**) SDS-PAGE of BdPepR2 WT, BdPepR2 M1066R, BdPepR2 M1066A. (**C**) LC–MS/MS quantification of cGMP levels generated by BdPepR2 recombinant and mutant proteins with Met1066 substituted with Arg or Ala in presence of various GTP concentrations and 5 mM MnCl_2_ after 15 min at 30 °C. Data are mean values (n = 3), and error bars show standard error of mean. Statistical analysis was performed by one-way ANOVA followed by a Tukey–Kramer multiple comparison test. ns indicates not significantly different data.

To investigate whether [M] at position 14 in GC center of Bd PepR2 is preferable, two mutants with different GC centers were created: BdPepR2M1066A and BdPepR2M1066R. This substitution is based on previous findings that indicated that the conversion from defined [KR] into [AL] causes a decrease in GC activity [26]. In result LC–MS/MS quantification of cGMP levels (as illustrated in Figure 5A,C) demonstrated that the BdPepR2M1066A protein manifests a significant reduction in cGMP production (22.199 pmol mg protein^−1^ min^−1^ (±10.070)) in comparison to that of the wild-type BdPepR2 recombinant protein (72.1 pmol mg protein^−1^ min^−1^ (±9.48)). However, the BdPepR2M1066R protein exhibited only a slight reduction in cGMP production at 57.774 pmol mg protein^−1^ min^−1^ (±5.692) (as illustrated in Figure 5A,C) which is consistent with the notion that a positively charged residue [KR] is required to interact with the phosphate of GTP in catalytic centers identified by the GC motif, as observed in the *A. thaliana* homolog AtPepR1-GC and other GCs. However, our results showed that in the case of BdPepR2, the neutral nonpolar [M] residue can assume the role of [KR] in that position and even exhibiting stronger activity, which could be a unique feature of monocot GCs. Previously identified as key residues [KR] are polar and have positively charged side groups, whereas [M] is a nonpolar amino acid, but docking stimulation showed that [M] at position 14 is pointing into the catalytic center and that it may be able to interact with the phosphate of GTP. While the BdPepR2M1066A variant confirmed previous findings, mutation of amino acid at position 14 to alanine of the AtBRI1-GC catalytic center resulted in decreased cGMP production [26] or chemically similar leucine due to unsuccessful docking stimulation [17,27]. Additionally, we analyzed the AC activity of the two mutant proteins and it was lower than that of the BdPepR2 WT (data not shown). This indicates that the mutation of amino acid at position 14 does not affect AC activity. A 14-amino-acid-long GC catalytic domain search motif was first deduced from the alignment of designated catalytic domains from vertebrates, lower eukaryotes, and prokaryotes. As mentioned earlier, there are no data on the GC activity of proteins with [M] at position 14 at the catalytic center in either guanylyl or ACs in plants or in other eukaryotes and prokaryotes. However, our data on BdPepR2 GC in vitro activity and structural analysis indicate that an expansion of the search motif should be considered. Since the GC center in BdPepR2 does not fit the GC motif used for the identification of GCs in *A. thaliana* and other dicots, the GC prediction tool GCPred [28] did not identify this protein as a candidate GC. Thus, our results suggest that the catalytic center may be wider than previously believed, and more monocot GCs should be identified to improve existing predictive tools and expand our current understanding of plant GCs. To date, core motifs were developed, and the in vitro GC activity was also confirmed primarily for *A. thaliana*, as it is a very well-known model plant. Notably, *B. distachyon* belongs to monocotyledonous plants, whereas most GCs with confirmed activity are from *A. thaliana*, a dicotyledonous plant. To the best of our knowledge BdPepR2 is only the third active GC that contain a highly conserved 14-aa search center in the GC catalytic site reported from monocotyledonous plants, after HpPepR1 [13], and OsWAKL21.2 [14]. Determining whether the change from [KR] to [M] is unique to monocots warrants further research. Alignment of the BdPepR2 aa sequence with other predicted leucine-rich repeat receptor-like protein kinase PEPR1/2 proteins from the *Poaceae* family showed identity in all 14 amino acids in the GC catalytic domain (as illustrated in Figure S1). It is likely that other proteins similar to the *B. distachyon* GC search motif may also have catalytic activity, thereby implying that a significant number of GCs remain to be identified, especially in plants other than *A. thaliana* and dicots.

### 2.4. BdPepR2 Kinase Activity

**Figure 6:**
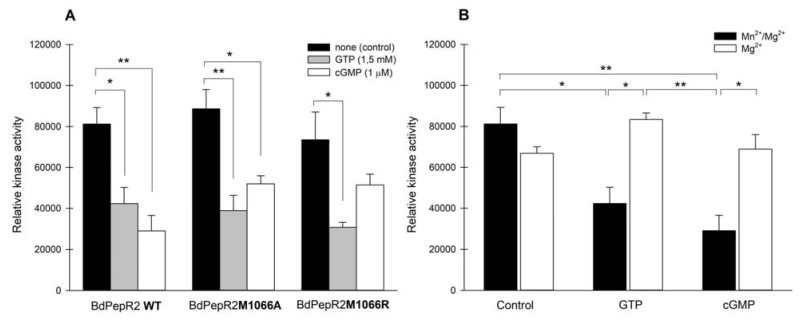
Determination of kinase activity of recombinant BdPepR2 by Kinase–Glo plus luminescent kinase assay. (**A**) Determination of kinase activity of recombinant BdPepR2 WT, BdPepR2 M1066A, and BdPepR2 M1066R and effect of cGMP or GTP on kinase activity. Reaction mixture contained 25 mM Tris/HCl buffer (pH 7.5), 5 mM MnCl_2_ and 5 mM MgCl_2_, 1 mM DTT, 1 μg/μL Histone Type III-S, 100 μM ATP (control), supplemented with 1 µM cGMP or 1.5 mM GTP and 1 μg of purified protein (without GST-tag) in a final volume of 50 μL. (**B**) Determining effect of GTP and addition of cofactors on kinase activity of BdPepR2 WT. Reaction mixture contained 25 mM Tris/HCl buffer (pH 7.5), 5 mM MgCl_2_ and/or 5 mM MnCl_2_, 1 mM DTT, 1 μg/μL Histone Type III-S, 100 μM ATP (control), supplemented with 1 µM cGMP or 1.5 mM GTP and 1 μg of purified protein (without GST-tag) in a final volume of 50 μL. Data are mean values (n = 3), and error bars show standard error of the mean. Statistical analysis was performed by one-way ANOVA followed by a Tukey–Kramer multiple comparison test (* *p* < 0.05; ** *p* < 0.01).

Using the Kinase-Glo plus luminescent kinase assay, we confirmed that BdPepR2 has serine/threonine kinase activity (as illustrated in Figure 6A). Moreover, we showed that all three proteins, BdPepR2, BdPepR2M1066A, and BdPepR2M1066R, have similar kinase activity. Both mutations had no effect on kinase activity, meaning that the amino acid at position 14 in GC center of Bd PepR2 does not affect the kinetic activity of protein, which suggests that the main function of this 14-aa-long region in the kinase domain is cGMP production. This is comparable to AtBRI1 [8] and AtPSKR1 [9] in which mutations in the GC catalytic center reduce cGMP generation but do not significantly change kinase activity. Moreover, we observe that kinase activity of BdPepR2, BdPepR2M1066A, and BdPepR2M1066R is suppressed by GTP and cGMP (as illustrated in Figure 6A). To check whether only cGMP or also GTP inhibits the kinase activity, we prepared an experiment with different components of the reaction buffer. The magnesium ions alone or magnesium and manganese ions together (manganese is necessary cofactor for the GC activity of the BdPepR2) were added. Interestingly, we observe that only the product of GC activity, cGMP, statistically significantly suppress kinase activity of BdPepR2 in vitro, and under conditions where cGMP formation is inefficient (only magnesium), the kinase activity was as high as in the control (as illustrated in Figure 6B). A similar effect of suppressing kinase activity by cGMP was observed on the kinase activity of AtBRI1 [8] and AtPSKR1 [9]. This results confirm that in addition to a functional GC domain, the recombinant BdPepR2 also harbors a functional kinase domain, thus making it a twin-domain molecule; moreover, cGMP suppresses kinase activity.

## 3. Materials and Methods

### 3.1. Construction of Expression Vectors

Total RNA was extracted from three-week-old *Brachypodium distachyon* Bd21 seedlings using a Universal RNA Purification Kit (EURx, Gdańsk, Poland). cDNA was synthesized using the NG dART RT kit (EURx, Gdańsk, Poland). A 942-bp fragment of BdPepR2 (NCBI accession number: XM_003571605.3; https://www.ncbi.nlm.nih.gov/nuccore; accessed on 27 March 2018) coding region corresponding to a 313-residue polypeptide [Met834→Lys1146] was amplified by PCR using specific primers (as illustrated in Table S1). Next, the PCR product was introduced into the linearized pGEX-6P-2 expression vector using In-Fusion cloning technology (In-Fusion HD Cloning Kit; Takara Bio USA, Inc., Mountain View, CA, USA). The *E. coli* BL21 strain, after being transformed with the resulting plasmid, was used to produce the glutathione S-transferase (GST)-tagged protein. BdPepR2 mutants (M1066A and M1066R) were generated using a QuikChange XL Site-Directed Mutagenesis Kit (Agilent, Santa Clara, CA, USA). pGEX-6P-2:BdPepR2 was used as a template, and in each case, mutagenesis primers were designed through https://www.agilent.com/store/primerDesignProgram.jsp (accessed on 20 November 2020) the relevant forward and reverse oligonucleotides are listed in Table S1). Each plasmid was verified through sequencing.

### 3.2. Expression and Purification of the Recombinant Proteins

For the expression of BdPepR2, BdPepR2M1066A, and BdPepR2M1066R recombinant proteins, the appropriate construct was transformed into One Shot BL21 (DE3)pLysS *E. coli* cells (Life Technologies, Carlsbad, CA, USA). Bacterial cells were grown in LB medium supplemented with 2% glucose at 37 °C. Fusion protein production was induced by adding IPTG at a final concentration of 1 mM and incubating the cells at 24 °C for 3 h in glass vessels connected to a BioFlo 120 bioprocess control station (Eppendorf, Hauppauge, NY, USA). The pH was maintained at 6.5 (±0.2), the dissolved oxygen parameter was set to 20%, and the agitation speed was 200 rpm. Bacteria were collected by centrifugation, and proteins were purified as previously described [13]. Proteins were released from the fusion protein by proteolytic cleavage of the protein with PreScission protease (GE Healthcare Europe GmbH, Freiburg, Germany) following the manufacturer’s instructions. The homogeneity and purity of the protein fractions were analyzed with 10% (*v*/*v*) SDS/PAGE. Pure protein, without a GST tag, was used for the activity analyzes.

### 3.3. Determination of Guanylyl Cyclase Activity

The guanylyl cyclase activity was determined by estimating the rate of cGMP formation. For the enzymatic assay, the reaction mixture contained 50 mM Tris/HCl buffer (pH 7.5), 5 mM MnCl_2_ or/and 5 mM MgCl_2_, GTP as a substrate (0.5–2 mM), and 5 μg of the purified protein in a final volume of 100 μL. After incubation at 30 °C for 15 min, the reaction was stopped at 100 °C for 10 min, and the samples were centrifuged at 16,000 *g* for 10 min. Moreover, ATP (1.5 mM) was used to analyze the specificity of the enzyme, and background cGMP levels were measured in tubes that contained the reaction mixture but no protein. The total cGMP concentration was determined in triplicate using liquid chromatography–tandem mass spectrometry (LC-MS/MS Nexera UHPLC and LCMS-8045 integrated system (Shimadzu Corporation, Kyoto, Japan)). The ionization source parameters were optimized in positive ESI mode using pure cGMP dissolved in HPLC-grade water (Sigma). Samples were separated using a reversed-phase C18 column (150 × 2.1 mm, 2.6 µm, Kinetex) in 10% methanol with 0.1% (*v*/*v*) formic acid (solvent A (water with 0.1% (*v*/*v*) formic acid), solvent B methanol with 0.1% (*v*/*v*) formic acid) at a flow rate of 0.3 mL/min. The interface voltage was set at 4.0 kV for positive (ES+) electrospray. Data acquisition and analysis were performed with the LabSolutions (Shimadzu Corporation, Kyoto, Japan) workstation for LCMS-8045. The enzyme activity was defined as the amount of cGMP produced by 1 mg of protein per min.

### 3.4. Determination of Proteins Kinase Activity

For quantification of BdPepR2 protein kinase activity, a Kinase-Glo plus luminescent kinase assay (V3771, Promega, Walldorf, Germany) was used according to the manufacturer’s protocol. Briefly, purified truncated BdPepR2, BdPepR2 M1066A or BdPepR2 M1066R (1 μg) was added to a 50-μL reaction mixture (25 mM Tris/HCl, pH 7.5, 5 mM MgCl_2_ or/and 5 mM MnCl_2_, 1 mM DTT, 1 μg/μL Histone Type III-S, and 100 μM ATP) supplied with 1 µM cGMP or 1.5 mM GTP. Reactions were performed at 30 °C for 15 min and stopped by adding the Kinase-Glo reagent. After equilibrating the mixture at room temperature for 10 min, luminescence was monitored using a Synergy HT Multi-Mode Microplate Reader. The results are expressed in relative luminesce units; experiments were undertaken in triplicate.

### 3.5. Computational Modeling

Full-length amino acid sequences of BdPerR2 (NCBI accession number: XM_003571605.3; https://www.ncbi.nlm.nih.gov/protein/), AtPepR1 (NCBI accession number: OAP14914.1; https://www.ncbi.nlm.nih.gov/protein/), and AtPepR2 (NCBI accession number: OAP12577.1; https://www.ncbi.nlm.nih.gov/protein/) were aligned using the Clustal Omega program. Domain predictions were performed using InterPro (https://www.ebi.ac.uk/interpro/) and LRRfinder (http://www.lrrfinder.com/lrrfinder.php). All web sites in this paragraph were accessed on 9 January 2021. The Met834→Lys1146 fragment of the BdPepR2 structure was modeled against the AtBRI1 template as described in [2] using MODELLER (ver. 9.25) software [29,30,31]. Docking simulations of GTP to the GC domain of BdPePR2 were performed using AutoDock Vina (ver. 1.1.2) [32]. All structures, binding poses, and images were analyzed and created using UCSF Chimera (ver. 1.10.1) [33].

## 4. Conclusions

The search for motifs and/or individual amino acids led to the analysis of the active centers of enzymes and the identification of new elements in the functional centers. Our successful change in amino acids provides strong validation of BdPepR2 activity. Thus, this result clarifies the next element in guanylyl cyclase activity and catalytic motif-site details. In this manuscript, we propose an explanation for the existence of other amino acids that are involved in cyclase activity. We identify that a motif with methionine, never before tested amino acid in cyclases, functions better than the conserved amino acids described previously; this motif binds to the phosphate acyl group and stabilizes the transition state from GTP to cGMP. So far, little is known about GCs in monocots plants. It is possible that the [M] at position 14 instead of [KR] is unique to monocots, and if so, more monocot candidates need to be examined to determine how the GCs evolved between monocots and dicots. Despite the importance of this discovery, it is still only one step in characterizing this group of plant proteins. Further studies will be necessary to compare the mechanisms governing the regulation of the enzyme, analyze their role in plant cells by transgenic plant production, and compare them to those of other GCs. Moreover, it would be important to define more closely the interactions among cyclase motif and kinase domain.

## Data Availability

Data is contained within the article or Supplementary Material.

## Supplementary Materials

The following are available online at https://www.mdpi.com/article/10.3390/ijms22126243/s1, Figure S1: The amino acid sequence alignment of predicted leucine-rich repeat receptor-like protein kinase PEPR1/2 proteins from the *Poaceae* family, Table S1: Sequence of the primers employed for PCR amplification in this study.
