# Supplementary material for: In Vitro Characterization of Guanylyl Cyclase BdPepR2 from Brachypodium distachyon Identified through a Motif-Based Approach
Source: Int J Mol Sci. 2021 Jun 10;22(12):6243. doi: 10.3390/ijms22126243 (PMC8228174; doi:10.3390/ijms22126243)
Supplement: Supplementary file 1 [file ijms-22-06243-s001.zip › Supplementary data/Table S1.pdf]

Table S1 SPECIFIC PRIMERS SEQUENCES

Sequence of the primers employed for PCR amplification in this study. The table includes oligonucleotide sequences employed for cloning (lower case letters indicate the *Sal*I and *Not*I restriction sites, respectively) and site-directed mutagenesis

Primer nomenclature	5'-3' oligonucleotide sequence
BdPepR2 (for cloning)	
BdPepR2 forward	GAATTCCCGGgtcgacAATGCCTGGGCGCCTATCG
BdPepR2 reverse	AGTCACGATgcggccgcTTACTTGTCCATTCTCACCAAGACA
BdTTM3 (for site-directed mutagenesis)	
BdPepR2 M1066A forward	TGCAGAATCCACAGGCGCCTTCCTGCACAGGAGC
BdPepR2 M1066A reverse	GCTCCTGTGCAGGAAGGCGCCTGTGGATTCTGCA
BdPepR2 M1066R forward	ATCCACAGGCCTCTTCCTGCACAGGAGCTC
BdPepR2 M1066R reverse	GAGCTCCTGTGCAGGAAGAGGCCTGTGGAT
