# Supplementary material for: In Vitro Characterization of Guanylyl Cyclase BdPepR2 from Brachypodium distachyon Identified through a Motif-Based Approach
Source: Int J Mol Sci. 2021 Jun 10;22(12):6243. doi: 10.3390/ijms22126243 (PMC8228174; doi:10.3390/ijms22126243)
Supplement: Supplementary file 1 [file Figure S1.pdf › Supplementary data/Figure S1.pdf]

```
Brachypodium        NVGLILYEYMPEGTLFELLHERKPQVALGWMARHQIALGVAQGLSYLHQDCVPMIVHRDV     992
Hordeum             GAGLILYEYMPEGTLFELLHGRRPQVALGWTARHQIALGLAQGLSYLHQDCVPMIVHRDV     1060
Triticum            GAGLILYEYMPEGTLFELLHGRKPQVALGWTVRHQIALGLAEGLSYLHQDCVPMIVHRDV     993
Oryza               SVGLILYEYMPEGTLFELLHRRKPHAALDWTVRHQIAFGVAQGLSYLHHDCVPMIVHRDV     976
Panicum             SIGLILCEYMPEGTLFDLFHQRKPQVALDWMTRHQIALGIAQGLSYLHHDCVPMIVHRDV     966
Setaria             SVGLILCEYMPKGTLFDLLHQRKPQVALDWMIRHQIALGVAQGLSYLHHDCVPMIVHRDV     1001
                    . **** ****:****:*:* *:*:.**.*  *****:*:*:******:***********

Brachypodium        KSSNILMDVELVPKLTDFGMGKIVGDEDSDATVSVIVGTLGYIAPEHGYSTRLSEKSDVY     1052
Hordeum             KSNNVLMDADMVPKLADFGMGKIVGDEDADATVSVIVGTLGYIAPEHGYSTRLTEKSDVY     1120
Triticum            KSSNVLMDADMVPKLADFGMGKIVGDEDADATVSVIVGTLGYIAPEHGYSTRLTEKSDVY     1053
Oryza               KSSNILMDTELVPKLTDFGMGKIVEDDDLDATVSVVVGTLGYIAPEHGYYTRLTEKSDVY     1036
Panicum             KSSNILMDSELVPKLTDFGMGKIVHDEDADATVSVIVGTLGYIAPEHGYSTRLTEKSDVY     1026
Setaria             KSSNILMDTELVPKLTDFGMGKIVHDEDANATVSAVIGTLGYIAPEHGYSTRLTEKSDVY     1061
                    **.*:*** ::****:******** *:* :****.::************ ***:******

Brachypodium        SYGVVLLELLCRKMPVDSAFGDGVDIVTWMRSNLKQADHC-SVMSCLDEEIVYWPEDEQA     1111
Hordeum             SYGVVLLELLCRKMAVDPAFGDGVDIVTWMRSNPKQADRRPAAMSCLDEEIVYWPEDEQA     1180
Triticum            SYGVVLLELLCRKMAVDPAFGDGVDIVTWMRSNLKQADRRPAAMSCLDEEIVYWPEDEQA     1113
Oryza               SYGVVLLELLCRKMPVDPAFGDSVDIVTWMRSNLTQADRR-VIMECLDEEIMYWPEDEQA     1095
Panicum             SYGVVLLELLCRKMPVDPSFGDGVDIATWMRTKLKQADHS-SIISLMDEEIMYWPEDEQE     1085
Setaria             SYGVVLLELLCRKMPVDPSFEDGVDIATWIRTKLKQADRC-SIIDLMDEEIMYWPEDDQE     1120
                    ************** ** :* *.***.**:*:: .***:    :. :****:*****:*

Brachypodium        KALHLLDLAISCTEVACQLRPSMREVVNVLVRMDK----     1146
Hordeum             RALDMLDLAISCTQFSFQSRPSMREVVNTLLRMDM----     1215
Triticum            KALDLLDLAISCTQAACQSRPSMREVVNTLVRMDMQGFP     1152
Oryza               KALDLLDLAMYCTQLACQSRPSMREVVNNLMRMDK----     1130
Panicum             KALDLLDLAVSCSQVACQSRPSMREVVNMLMKIEK----     1120
Setaria             KALDLLDLAVSCTQVACQSRPSMREVVNMLLKIEK----     1155
                    :**.:****: *:: : * ********* *::::
```

Figure S1. Alignment of the BdPepR2 aa sequence with other predicted leucine-rich repeat receptor-like protein kinase PEPR1/2 proteins from the *Poaceae* family.

XP_003571653.2 leucine-rich repeat receptor-like protein kinase PEPR2 [Brachypodium distachyon]

KAE8784640.1 Receptor-like protein kinase [Hordeum vulgare]

XP_037434473.1 leucine-rich repeat receptor-like protein kinase PEPR2 [Triticum dicoccoides]

XP_015612899.1 leucine-rich repeat receptor-like protein kinase PEPR2 [Oryza sativa Japonica Group]

XP_025797572.1 leucine-rich repeat receptor-like protein kinase PEPR1 isoform X1 [Panicum hallii]

XP_004983380.2 leucine-rich repeat receptor-like protein kinase PEPR1 [Setaria italica]
